# Electrochemically Pretreated Sensor Based on Screen-Printed Carbon Modified with Pb Nanoparticles for Determination of Testosterone

**DOI:** 10.3390/ma15144948

**Published:** 2022-07-15

**Authors:** Jędrzej Kozak, Katarzyna Tyszczuk-Rotko, Magdalena Wójciak, Ireneusz Sowa, Marek Rotko

**Affiliations:** 1 Institute of Chemical Sciences, Faculty of Chemistry, Maria Curie-Skłodowska University in Lublin, 20-031 Lublin, Poland; jedrekkozak@onet.pl (J.K.); marek.rotko@poczta.umcs.lublin.pl (M.R.); 2 Department of Analytical Chemistry, Medical University of Lublin, 20-093 Lublin, Poland; magdalena.wojciak@umlub.pl (M.W.); i.sowa@umlub.pl (I.S.)

**Keywords:** testosterone, electrochemical preparation, screen-printed carbon sensor, lead nanoparticles, differential-pulse adsorptive stripping voltammetry, human urine, wastewater

## Abstract

Testosterone (TST), despite its good properties, may be harmful to the human organism and the environment. Therefore, monitoring biological fluids and environmental samples is important. An electrochemically pretreated screen-printed carbon sensor modified with Pb nanoparticles (pSPCE/PbNPs) was successfully prepared and used for the determination of TST. The surface morphology and electrochemical properties of unmodified and modified sensors were characterized by cyclic voltammetry (CV), electrochemical impedance spectroscopy (EIS), scanning and transmission electron microscopy (SEM and TEM), and energy-dispersive X-ray spectroscopy (EDS). Selective determinations of TST at the pSPCE/PbNPs were carried out by differential pulse adsorptive stripping voltammetry (DPAdSV, E_Pb dep.and TST acc._ of −1.1 V, t _Pb dep.and TST acc._ of 120 s, ΔE_A_ of 50 mV, ν of 175 mV s^−1^, and t_m_ of 5 ms) in a solution containing 0.075 mol L^−1^ acetate buffer of pH = 4.6 ± 0.1, and 7.5 × 10^−5^ mol L^−1^ Pb(NO_3_)_2_. The analytical signal obtained at the potential around −1.42 V (vs. silver pseudo-reference electrode) is related to the reduction process of TST adsorbed onto the electrode surface. The use of pSPCE/PbNPs allows obtaining a very low limit of TST detection (2.2 × 10^−12^ mol L^−1^) and wide linear ranges of the calibration graph (1.0 × 10^−11^–1.0 × 10^−10^, 1.0 × 10^−10^–2.0 × 10^−9^, and 2.0 × 10^−9^–2.0 × 10^−8^ mol L^−1^). The pSPCE/PbNPs were successfully applied for the determination of TST in reference material of human urine and wastewater purified in a sewage treatment plant without preliminary preparation.

## 1. Introduction

Hormones regulate many types of cellular and physiological functions in the human body, such as reproduction, growth, and differentiation [1]. Testosterone (TST), chemically known as 17β-hydroxyandrost-4-en-3-one, is the principal endogenous androgenic–anabolic steroid in humans. In the human body, it is produced primarily in the testes of males and in the ovaries of females, while small amounts are produced by adrenal glands in both sexes [2,3]. In men, TST plays a key role in the development of male reproductive tissues such as the testis and prostate, as well as in promoting secondary sexual characteristics such as increased muscle, bone mass, and the growth of body hair. Moreover, TST is essential for health and well-being as well as the prevention of osteoporosis. Testosterone abuse is widespread among sportsmen willing to increase aggressiveness, strength, and recovery, making it the most frequently reported substance in steroid misuse. The World Anti-Doping Agency prohibited its use to ensure fair play and protect athletes from possible adverse side effects such as heart attack, high blood pressure, liver disease, or mental effects [2,4]. TST can be an ingredient in pharmaceuticals. In the urine of an average man, TST is present at a level of 10^−8^ mol L^−1^, but in the case of hormone therapy using TST, these concentrations can be several times higher [5]. Currently, we are dealing with increasing pollution of the environment with various types of pharmaceuticals, including hormones. TST is one of the organic micropollutants present in the environment and in natural waters and can cause adverse biological effects on humans and wildlife below the physiological levels (sub-ng L^−1^) [6,7]. Due to the fact that TST concentrations detected in the environment are in the order of 10^−12^–10^−11^ mol L^−1^ (groundwater [8] and municipal wastewater [9]), it is necessary to develop highly sensitive methods of measuring this hormone.

Among the popular analytical methods used for the detection of TST, chromatographic methods can be indicated, e.g., high-performance liquid chromatography coupled with tandem mass spectrometry (HPLC-MS/MS) [10], isotope dilution ultra-performance liquid chromatography–tandem mass spectrometry (ID-UPLC-MS/MS) [11], liquid chromatography coupled with mass spectrometry (LC-MS) [12,13], and gas chromatography coupled with mass spectrometry (GC-MS) [14,15]. Other methods that allow us to determine TST are capillary electrophoresis (CE) [16,17] and the molecularly imprinted plasmon resonance method [18]. While chromatographic methods are extremely effective, most have many disadvantages, such as cost and long and complicated sample pretreatment, usually involving different types of derivatization, extraction, and purification prior to analysis.

On the other hand, electrochemical methods provide fast, low-cost on-site analysis with high specificity and high sensitivity [4,6]. However, there are only a few studies available on the voltammetric determination of testosterone. Most of them show the use of conventional working electrodes such as glassy carbon electrodes modified in various ways—modified with a lead film (PbFE) [19], a cationic surfactant (GCE/CTAB) [4], or a cationic surfactant and a bismuth film (GCE/CTAB/BiF) [3]. It can also include maltodextrin-modified paste electrodes based on various carbon materials (graphite, graphene, carbon nanotubes, and fullerene C_60_) [1], the hanging mercury drop electrode (HMDE) [20], the edge plane pyrolytic graphite electrode modified with single-walled carbon nanotubes (SWNTs-EPPGE) [2], and a gold electrode modified with a double-layered molecularly imprinted polymer (AuE/DMIP) [21]. The lowest detection limit at the conventional working electrode, equal to 1.0 × 10^−14^ mol L^−1^, was obtained on the AuE/DMIP. However, the preparation of this electrode requires many reagents and a multi-step procedure consisting of cleaning the gold surface and electrodepositing the first conductive polymer layer, and then another one forming the DMIP. The final step is to remove the testosterone template and dry the electrode.

Unlike individual working electrodes in electrochemical analysis, all electrodes of screen-printed sensors (SPEs), i.e., reference, working, and counter electrodes, are printed and integrated on the same substrate. SPEs represent a modern analytical chemistry trend in miniaturization [22,23]. Screen-printed electrodes have advantages such as simplicity of construction and operation, diversification of the selection of electrode materials, low cost, design flexibility, reliability for detecting different substances, portability, and simplicity of modification of the electrodes for various uses [24]. An SPE is a good electrode due to its mass production, low cost, and low background current [25]. Conductive inks from screen-printed carbon electrodes (SPCEs) contain carbon with organic solvents, bonding pastes (e.g., polyester resin, ethyl cellulose, or epoxy-based polymer binder), and some additives that provide functional properties. The presence of these additional non-conductive materials can lead to a slowdown in the kinetics of heterogeneous electrochemical reactions [26]. The main purpose of the SPCE pretreatment is to remove the organic components of the ink or contaminants and to increase the surface roughness or functionality [27]. The following methods of pretreatment of SPEs can be found in the literature—heat treatment [27], oxygen plasma treatment [28], chemical treatment [29], polishing [30,31], and electrochemical treatment [32,33,34].

Nanomaterials are chemical substances or materials that are manufactured and used at a very small scale [35]. Among the nanomaterials, carbon nanomaterials are often used today as electrode modifiers. We can distinguish here graphene, carbon black (CB), carbon nanofibers (CNFs), carbon nanotubes (CNTs), and carbon nanohorns (CNHs). Carbon nanomaterials have proven to be efficient electrode materials as they exhibit remarkable electronic, mechanical, and chemical properties; high surface areas; low electrical resistance; excellent electrical conductivity; and low cost. Additionally, the ability to functionalize their surfaces with antibodies, nucleic acids, or catalysts can lead to enhanced analytical performance, including sensitivity and selectivity [36,37,38]. Another group of commonly used nanomaterials is nanoparticles (NPs), mainly metal nanoparticles. Due to their small size, nanoparticles can increase the surface area of the electrode used. In addition, metallic nanoparticles can increase the mass transport speed and provide fast electron transfer between the electroactive species and the electrode surface, which increases the sensitivity of the electrodes used [39,40].

Only one study describes the determination of testosterone using screen-printed sensors [41]. The TST determination procedure presented in the article [41] used SPEs modified with molecularly imprinted polymer (MIP). A very low LOD was obtained on this electrode, equal to 3.5 × 10^−17^ mol L^−1^. However, the preparation of the SPE/MIP is laborious and time-consuming and requires steps such as electropolymerizing the MIP on the surface of the working electrode in the presence of a high concentration of TST as a template and then removing this template. Therefore, a very simple procedure for the preparation of the modified screen-printed sensor was proposed while maintaining the high sensitivity and selectivity of the sensor. In this work, the combination of the valuable properties of screen-printed carbon electrode (SPCE) and lead nanoparticles (PbNPs), as well as the electrochemical pretreatment step in the fabrication of a novel voltammetric sensor of TST, was proposed for the first time. The use of a lead film glassy carbon electrode for TST determination was described in the literature [19]. However, as far as we know, the application of an electrochemically pretreated screen-printed carbon sensor modified with Pb nanoparticles (pSPCE/PbNPs) has never been reported. Moreover, it is the first time a voltammetric sensor has been used in TST determinations not only in body fluids (urine) but also in environmental samples (wastewater). It is worth adding that the samples do not require preliminary preparation. To specify the advantages of PbNPs and the use of the electrochemical pretreatment step, the pSPCE/PbNPs were characterized by cyclic voltammetry (CV), electrochemical impedance spectroscopy (EIS), scanning and transmission electron microscopy (SEM and TEM), and energy-dispersive X-ray spectroscopy (EDS).

## 2. Materials and Methods

### 2.1. Apparatus

Transmission electron microscopy (TEM) analysis was performed by means of a high-resolution transmission electron microscope Tecnai G2 T20 X-TWIN (FEI) equipped with an energy dispersive X-ray spectrometer (EDS). The samples were prepared for analysis by scratching the film from the surface of the electrode and placing it on a TEM copper grid. Moreover, microscopic images of the pSPCE/PbNPs surface were attained with a high-resolution scanning electron microscope Quanta 3D FEG (FEI, USA) (acceleration voltage of 5.0 kV, working distance of 9.3 mm, magnification of 25,000×).

All voltammetric studies were made using a µAutolab electrochemical analyzer (Eco Chemie, Utrecht, The Netherlands) controlled by GPES 4.9 software. The standard quartz electrochemical cell with a volume of 10 mL composed of a commercially available screen-printed carbon sensor (SPCE, DropSens, Spain, Ref. C150) was applied for experiments. The SPCE sensor consisted of a screen-printed carbon working electrode, a platinum screen-printed auxiliary electrode, and a silver screen-printed pseudo-reference electrode. The µAutolab analyzer controlled by FRA 4.9 software was used for electrochemical impedance spectroscopy (EIS) measurements.

HPLC analyses were performed on a VWR Hitachi Elite LaChrom HPLC with a PDA detector using an Ascentis Express C18 column (15 cm × 2.1 mm i.d., 2.7 μm).

### 2.2. Reagents and Solutions

Appropriate amounts of Merck reagent (Darmstadt, Germany), testosterone propionate, were dissolved in ethanol to obtain a 10^−3^ mol L^−1^ solution of TST. This solution was diluted with ethanol to obtain a 10^−4^ mol L^−1^ solution of TST or with 0.1 mol L^−1^ acetate buffer of pH = 4.6 ± 0.1 to obtain 10^−5^ or 10^−6^ mol L^−1^ solutions of TST. The supporting electrolyte, acetate buffer of pH = 4.6 ± 0.1, was prepared with reagents (CH_3_COONa and CH_3_COOH) purchased from Merck. The 10^−3^ mol L^−1^ stock solutions of Fe(III), Ca(II), Cu(II), Mg(II), Cd(II), Ni(II), V(V), glucose (GL), dopamine (DA), ascorbic acid (AA), uric acid (UA), epinephrine (EP), and adenine (AD) were prepared from Merck reagents in deionized water before starting the set of experiments and stored at 4 °C in the dark until used. HPLC-grade acetonitrile was purchased from Merck. The solutions were prepared using ultra-purified water supplied by a Milli-Q system.

### 2.3. Fabrication of pSPCE/PbNPs and Voltammetric Determination of TST

**Figure 1:**
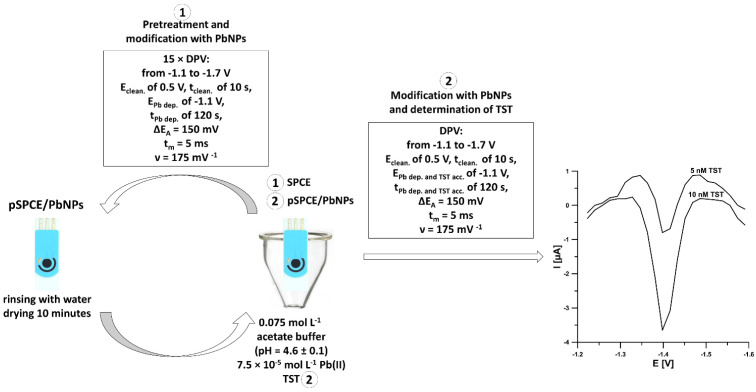
Scheme of sensor fabrication and voltammetric measurements of TST at the pSPCE/PbNPs.

The scheme of sensor fabrication and voltammetric measurements of TST at the pSPCE/PbNPs is presented in Figure 1. The commercially available SPCE was simultaneously electrochemically pretreated and electrochemically decorated by lead nanoparticles (PbNPs) in 0.075 mol L^−1^ acetate buffer of pH = 4.6 ± 0.1 containing 7.5 × 10^−5^ mol L^−1^ Pb(NO_3_)_2_. After placing a fresh electrode in the solution, 15 consecutive differential-pulse voltammograms were recorded (an electrochemical cleaning step at a potential of 0.5 V (E_clean._) for 10 s (t_clean._), modification of the surface with PbNPs at a potential of −1.1 V (E_Pb dep._) for 120 s (t_Pb dep._), a scan rate (ν) of 175 mV s^−1^, an amplitude (ΔE_A_) of 50 mV, a modulation time (t_m_) of 5 ms, and a differential-pulse scan from −1.1 to −1.7 V). Then, after rinsing the electrode with water, it was allowed to dry for 10 min at room temperature. The sensor was electrochemically pretreated only once before a series of measurements of TST.

The pSPCE/PbNPs fabricated were used for TST determination in the same solution (0.075 mol L^−1^ acetate buffer of pH = 4.6 ± 0.1 containing 7.5 × 10^−5^ mol L^−1^ Pb(NO_3_)_2_) in which it had been prepared. Only a specified amount of TST standard solution (concentration of TST in the range of 1.0 × 10^−11^–2.0 × 10^−8^ mol L^−1^) or sample was introduced into the supporting electrolyte. The procedure consists of an electrochemical cleaning step at a potential of 0.5 V (E_clean._) for 10 s (t_clean._), simultaneous modification of the surface with PbNPs, and accumulation of TST at a potential (E_Pb dep. and TST acc._) of −1.1 V for a time (t_Pb dep. and TST acc._) of 120 s. Differential-pulse scans were registered from −1.1 to −1.7 V with ν of 250 mV s^−1^, ΔE_A_ of 150 mV, and t_m_ of 5 ms.

### 2.4. HPLC/PDA Analysis

Chromatographic conditions were established based on the literature [42] with slight modification. A mixture of acetonitrile and water (65:35 *v*/*v*) at a flow rate of 0.25 mL min^−1^ was used as the mobile phase. The temperature was set at 30 °C. The injection volume was 10 µL, and the analytical wavelength was 240 nm.

### 2.5. Sample Analysis

The reference material of human urine (Medidrug Basis-line U) and wastewater purified in a sewage treatment plant (Lublin, Poland) were analyzed using the DPAdSV and HPLC/PDA methods. The desired concentrations of TST were added to the samples, and they were directly analyzed without any separation steps.

## 3. Results and Discussion

### 3.1. Characteristics of Sensors

**Figure 2:**
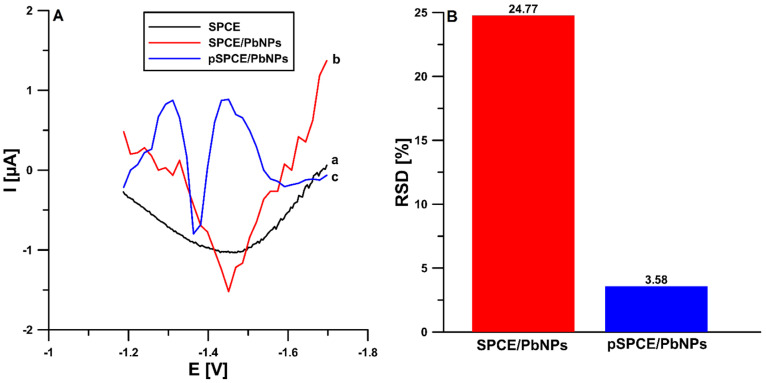
(**A**) DPAdSV curves of 2 × 10^−9^ mol L^−1^ TST recorded at the unmodified SPCE (**a**), modified with PbNPs (**b**), and the electrochemically pretreated SPCE/PbNPs (**c**). (**B**) Histogram bars of the repeatability of the TST signal (relative standard deviation (RSD), 2 × 10^−9^ mol L^−1^ TST, *n* = 10) at the SPCE/PbNPs and pSPCE/PbNPs.

In the first phase of the research, the differential-pulse adsorptive stripping voltammetry (DPAdSV) technique was used to characterize TST behavior at the pSPCE/PbNPs sensor. The studies were performed in 0.1 mol L^−1^ acetate buffer of pH equal to 4.6 ± 0.1 containing 7.5 × 10^−5^ mol L^−1^ Pb(NO_3_)_2_ and 2.0 × 10^−9^ mol L^−1^ TST. For comparison, the DPAdSV curves were recorded under the same conditions at the unmodified SPCE and the SPCE/PbNPs that was not electrochemically pretreated. The studies (Figure 2A) showed that the use of modification with lead nanoparticles was necessary to obtain a reduction in the TST signal. Moreover, the application of electrochemical pretreatment of the SPCE (15 consecutive DPV measurements: 0.5 V for 10 s, −1.1 V for 120 s, scan from −1.1 to −1.7 V in the solution used further for TST determinations, rinsing with water and drying for 10 min) practically does not change the TST peak current (1.80 vs. 1.74 µA), but significantly improves its shape and shifts the peak potential of TST towards less negative potential values (−1.45 vs. −1.36 V). Furthermore, the electrochemical pretreatment significantly improves the repeatability of the analytical signal (Figure 2B, 2.0 × 10^−9^ mol L^−1^ TST RSD of 24.77 vs. 3.58%, *n* = 10). In summary, the electrochemical pretreatment step was crucial for a nicely shaped and repeatable signal, which has already been described in the literature [33]. It is worth adding that in contrast to the works described so far [43], in the electrochemical pretreatment step, the same solution and parameters as for the TST determination were used, which simplifies the electrode preparation step and reduces the consumption of reagents.

**Figure 3:**
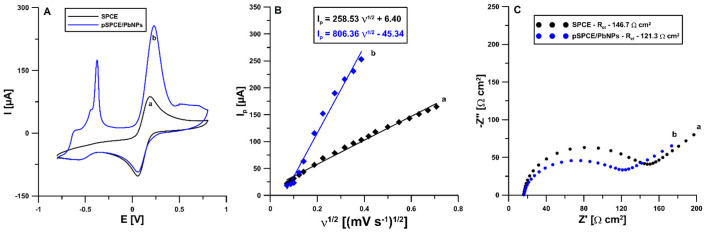
(**A**) Cyclic voltammograms recorded at the SPCE (**a**) and pSPCE/PbNPs (**b**) using the scan rate of 100 mV s^−1^; (**B**) the relationship between the anodic peak current (I_p_) and the square root of the scan rate (υ^1/2^) obtained at the SPCE (**a**) and pSPCE/PbNPs using the scan rate from 5 to 150 mV s^−1^; (**C**) Nyquist plots of the SPCE (**a**) and pSPCE/PbNPs (**b**) registered at a potential of 0.2 V, in the frequency range from 50 kHz to 1 Hz. All results were performed in 0.1 mol L^−1^ KCl and 5.0 mmol L^−1^ K_3_(Fe(CN)_6_).

The interfacial electron transport ability of the unmodified SPCE and the electrochemically pretreated SPCE/PbNPs was studied using EIS and CV techniques in 0.1 mol L^−1^ KCl containing 5.0 mmol L^−1^ K_3_(Fe(CN)_6_). The CV curve displayed a pair of well-defined redox peaks of (Fe(CN)_6_)^3-/4-^ at the unmodified SPCE (Figure 3A, curve a). In the case of the pSPCE/PbNPs (Figure 3A, curve b), the peak-to-peak separation (ΔE) increases from 123.6 to 169.0 mV, which is ascribed to the inhibition of the electrochemical reaction process by the PbNPs modification and electrochemical pretreatment. Moreover, the rate of the electron transfer at the SPCE and the pSPCE/PbNPs was calculated as the relative peak separations (χ^0^) by dividing ΔE by 59 mV. The χ^0^ values for the SPCE and pSPCE/PbNPs were greater than the theoretical value (χ^0^ = 1) and were equal to 2.09 and 2.86, respectively. Furthermore, the pSPCE/PbNPs show a higher anodic current intensity than the SPCE. The new peak at a potential around −0.5 V is related to the oxidation of lead from the pSPCE/PbNPs surface. The obtained results indicate that the PbNPs modification and electrochemical pretreatment inhibit the electron transfer kinetics. In addition, the Randles–Sevcik equation, CV curves recorded at scan rates of 5–150 mV s^−1^, and the dependence between the anodic peak current (I_p_) and the square root of the scan rate (v^1/2^) (Figure 3B) were used to calculate of the electrochemically active electrode area (A_s_) of the SPCE and pSPCE/PbNPs [44]. The A_s_ values of the SPCE and pSPCE/PbNPs were calculated to be 0.072 and 0.22 cm^2^, respectively. It is evident that the PbNPs modification and electrochemical pretreatment significantly increase the A_s_. Moreover, the impedance spectra (Nyquist plots) were recorded at the SPCE and pSPCE/PbNPs in the frequency range from 50 kHz to 1 Hz (Figure 3C). According to the experimental results, the charge transfer resistance (R_ct_) values obtained for the SPCE and pSPCE/PbNPs are 146.7 and 121.3 Ω, respectively. The pSPCE/PbNPs are characterized by lower R_ct_ and good conductivity.

**Figure 4:**
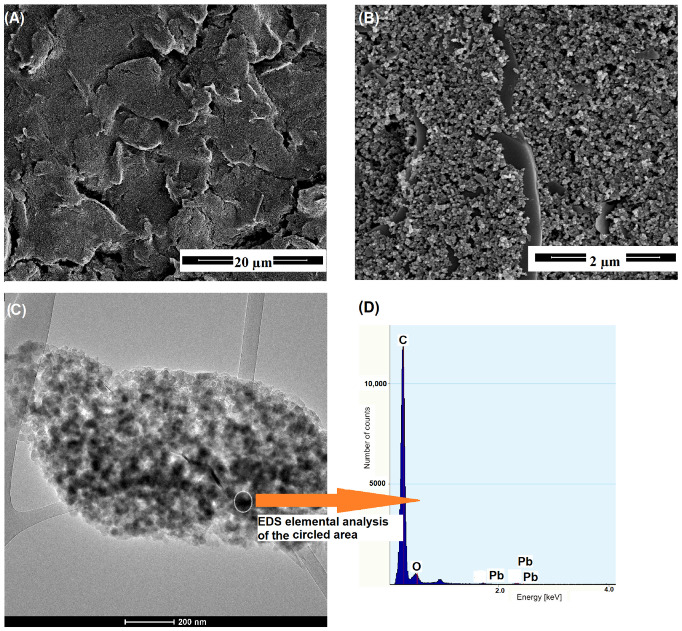
The SEM (**A**,**B**) and TEM (**C**) images of the pSPCE/PbNPs surface. (**D**) The EDS spectrum of the highlighted fragment of the pSPCE/PbNPs. The concentration of Pb(NO_3_)_2_ was 7.5 × 10^−5^ mol L^−1^.

In order to specify the advantages of PbNPs and the use of the electrochemical pretreatment step, the pSPCE/PbNPs were also characterized by scanning and transmission electron microscopy (SEM and TEM) and energy-dispersive X-ray spectroscopy (EDS). The SEM image of the pSPCE/PbNPs shows cracks formed during the drying of the SPCE surface (Figure 4A). Moreover, the characteristic structure of the carbon layer obtained by the screen-printing technique is visible in the higher resolution SEM image (Figure 4B). However, the presence of electrochemically deposited lead nanoparticles (PbNPs) was only detected using a high-resolution transmission microscope equipped with an energy dispersive X-ray spectrometer (EDS) (Figure 4C,D). The EDS analysis confirms that the black dots contain very small amounts of lead (mass % = 0.11), which confirms that the electrochemically deposited lead is rewarded in the form of nanoparticles.

### 3.2. Mechanism and Optimization Procedure

**Figure 5:**
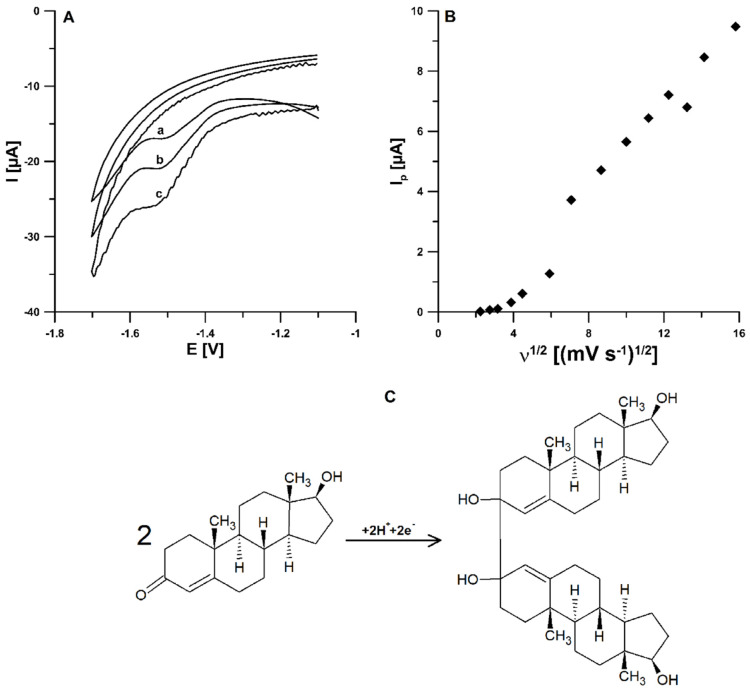
(**A**) CVs obtained at the pSPCE/PbNPs in the 0.075 mol L^−1^ acetate buffer of pH 4.6 ± 0.1 containing 7.5 × 10^−5^ mol L^−1^ Pb(NO_3_)_2_ and 5.0 × 10^−6^ mol L^−1^ TST (υ of 35, 50, 75 mV s^−1^). (**B**) The dependence between TST signal (I_p_) and the square root of the scan rate (υ) (υ in the range of 5–250 mV s^−1^). (**C**) The possible TST reduction mechanism.

In order to identify the involved TST reduction mechanism at the pSPCE/PbNPs, the effect of scan rate was investigated. The cyclic voltammograms of 0.075 mol L^−1^ acetate buffer of pH ± 0.1 containing 7.5 × 10^−5^ mol L^−1^ Pb(NO_3_)_2_ and 5.0 × 10^−6^ mol L^−1^ TST were recorded at scan rates from 5 to 250 mV s^−1^. Figure 5A demonstrates the CVs obtained for three scan rate values (35, 50, and 75 mV s^−1^). There is a cathodic peak and no anodic peak in the CVs of TST, indicating an irreversible electrode process. The TST reduction mechanism (Figure 5C) is well described in the literature [3]. It shows that the electrode process for TST is two-proton coupled two-electron transfer. As can be seen in Figure 5B, the TST signal (I_p_) increases non-linearly with the square root of the scan rate (υ). The non-linear I_p_/υ plot with the regression equation of I_p_ (µA) = 0.74 × υ^1/2^ ((mV s^−1^)^1/2^) − 2.17 indicates that the faradic reaction is controlled by an adsorption process.

**Figure 6:**
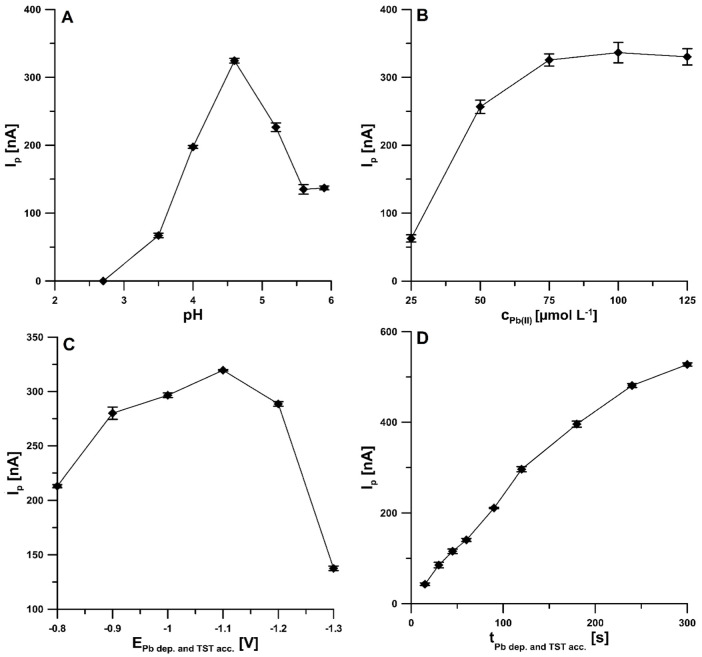
The dependence of pH (**A**), Pb(NO_3_)_2_ concentration (**B**), E_Pb dep. and TST acc._ (**C**), and t_Pb dep. and TST acc._ (**D**) on 1 × 10^−8^ mol L^−1^ TST signal. The DPAdSV parameters: t_m_ of 10 ms, ΔE_A_ of 50 mV and ν of 40 mV s^−1^. The mean values of I_p_ are given with the standard deviation for *n* = 3.

Additionally, the effect of pH value (acetic acid and acetate buffer) on the reduction peak current of 1.0 × 10^−8^ mol L^−1^ TST was studied. The progress of I_p_ with pH shows that (Figure 6A) this parameter increased up to pH 4.6 ± 0.1, and therefore, an acetate buffer of pH 4.6 ± 0.1 was selected for further studies. Furthermore, the TST reduction process was analyzed at various concentrations (from 0.025 to 0.125 mol L^−1^) of acetate buffer (pH 4.6 ± 0.1) at the pSPCE/PbNPs. The fixed concentration of TST (1.0 × 10^−8^ mol L^−1^) was added to the supporting electrolyte. According to the results, the highest peak current was obtained at an acetate buffer concentration of 0.075 mol L^−1^. Then, the effect of Pb(NO_3_)_2_ concentration was evaluated in the range of 2.5 × 10^−5^ to 1.25 × 10^−4^ mol L^−1^ towards the reduction peak current of 1.0 × 10^−8^ mol L^−1^ TST. As exposed in Figure 6B, when increasing the Pb(NO_3_)_2_ concentration, the TST response also increases up to 7.5 × 10^−5^ mol L^−1,^ and therefore, this concentration value was chosen. Moreover, the impact of DPAdSV procedure parameters, such as simultaneous modification of the surface with PbNPs and accumulation of TST potential (E_Pb dep. and TST acc._) and time (t_Pb dep. and TST acc._), amplitude (ΔE_A_), scan rate (ν), and modulation time (t_m_), on the peak currents of 1.0 × 10^−8^ mol L^−1^ TST was investigated. The E_Pb dep. and TST acc._ were tested in the range from −0.8 to −1.3 V. The results (Figure 6C) show that the highest TST signal was obtained for −1.1 V (t_Pb dep. and TST acc._ was equal to 120 s), and hence this value was chosen as optimal. Next, for the selected value of the potential, the effect of t_Pb dep. and TST acc._ in the range of 15–300 s was examined. The t_Pb dep. and TST acc._ of 120 s was selected for further study (Figure 6D), but the stage of simultaneous modification of the surface with PbNPs and accumulation of TST can be extended to obtain lower detection limits.

**Figure 7:**
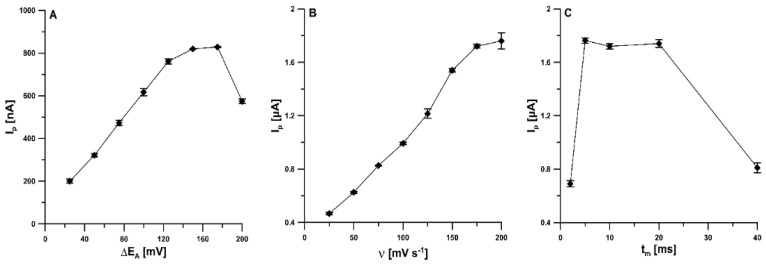
The dependence of ΔE_A_ (**A**), ν (**B**), and t_m_ (**C**) on 1 × 10^−8^ mol L^−1^ TST signal. The DPAdSV parameters: E_Pb dep. and TST acc._ of −1.1 V and E_Pb dep. and TST acc._ of 120 s. The mean values of I_p_ are given with the standard deviation for *n* = 3.

In order to investigate the effect of ΔE_A_ (from 25 to 200 mV), the reduction peak current of TST was measured (Figure 7A). The best responses were obtained with ΔE_A_ of 150 and 175 mV. For further studies, the value of 150 mV was chosen. Figure 7B depicts the effect of ν in the range of 25–200 mV s^−1^ on the TST signal. The TST reduction signal increased by increasing υ up to 200 mV s^−1^. Due to the better repeatability of the TST signal, υ of 175 mV s^−1^ was selected as optimal. The t_m_ was checked in the range from 2 to 40 ms. The highest TST signal was recorded for the t_m_ of 5 ms (Figure 7C).

### 3.3. Voltammetric Determination of TST

**Figure 8:**
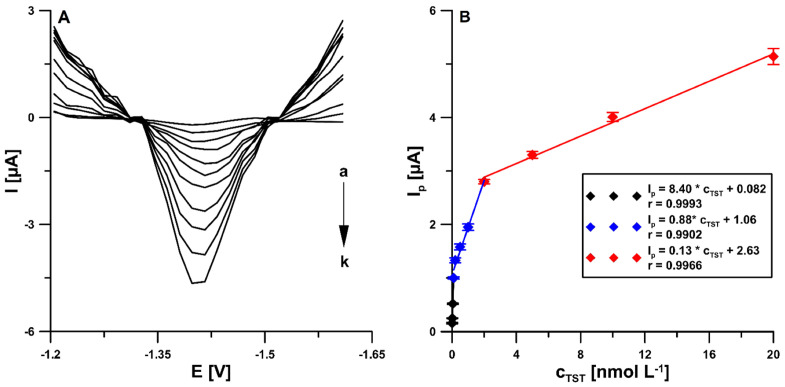
The DPAdSVs of the pSPCE/PbNPs in the presence of various TST concentrations (a → k, 1.0 × 10^−11^–2.0 × 10^−8^ mol L^−1^) in 0.075 mol L^−1^ acetate buffer of pH 4.6 ± 0.1 and 7.5 × 10^−5^ mol L^−1^ Pb(NO_3_)_2_ (**A**).Calibraion graph of TST (**B**). The obtained average values of the peak current are shown with standard deviation for *n* = 3. The DPAdSV parameters: t_m_ of 5 ms, ΔE_A_ of 150 mV, ν of 175 mV s^−1^, E_Pb dep. and TST acc._ of −1.1 V and E_Pb dep. and TST acc._ of 120 s.

The determination of TST at different concentrations was performed at the pSPCE/PbNPs by the DPAdSV technique under the developed conditions. Figure 8 shows the obtained results. A_s_ the concentration of TST increased, the related reduction peak current also increased. The plot of the peak current against TST concentration exhibited three linear ranges. The first one was from 1.0 × 10^−11^ to 1.0 × 10^−10^ mol L^−1^, the second one was from 1.0 × 10^−10^ to 2.0 × 10^−9^ mol L^−1^, and the third one was from 2.0 × 10^−9^ to 2.0 × 10^−8^ mol L^−1^. The detection (LOD) and quantification (LOQ) limits were estimated to be 2.2 × 10^−12^ and 7.3 × 10^−12^ mol L^−1^, respectively, using LOD = 3SD_a_/b and LOQ = 10 SD_a_/b equations (SD_a_—standard deviation of intercept (*n* = 3); b—slope of calibration curve) [45].

The linear range and the LOD of the pSPCE/PbNPs were compared with other previously reported voltammetric sensors, and the data are presented in Table 1. It can be seen that only two studies describe the determination of TST with a lower LOD [21,41]. However, the preparation of these electrodes (SPEs modified with a molecularly imprinted polymer and AuE modified with a double-layered molecularly imprinted polymer) requires a more expensive apparatus; the procedures are more labor-intensive, and more chemicals are used.

**Figure 9:**
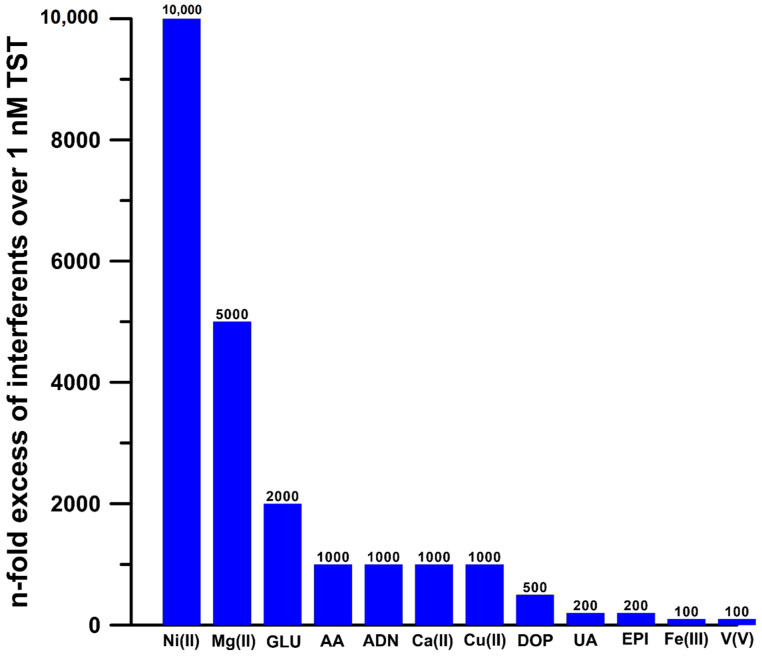
Histogram of the selectivity of pSPCE/PbNPs for TST determination. GLU—glucose, AA—ascorbic acid, ADN—adenine, DOP—dopamine, UA—uric acid, EPI—epinephrine.

In order to investigate the selectivity of the DPAdSV procedure with the use of the pSPCE/PbNPs for TST determination, increasing concentrations of potential interferents were added to the supporting electrolyte. The tolerance limit was defined as the concentration that gave an error of ≤10% in the determination of 1.0 × 10^−9^ mol L^−1^ TST. It was noted that studied substances have negligible effects on the peak current of TST (Figure 9).

### 3.4. TST Determination in Real Samples

**Figure 10:**
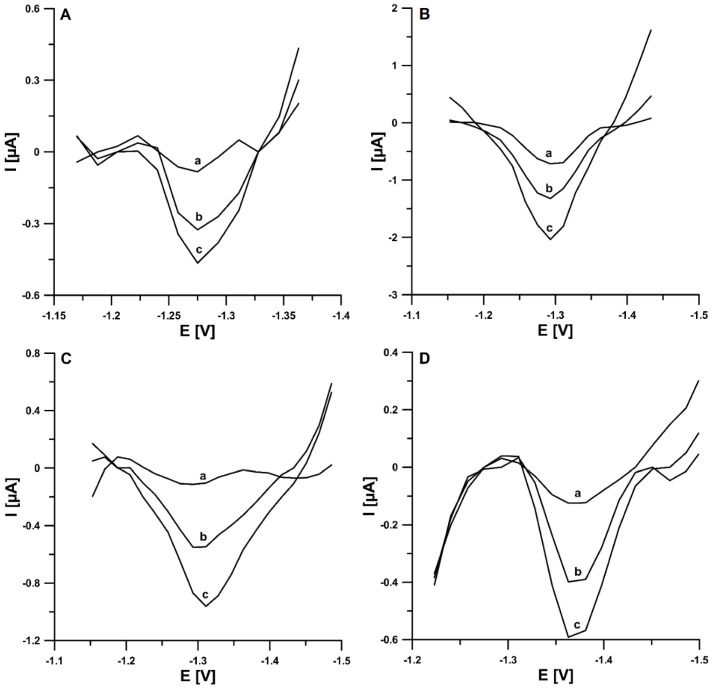
The DPAdSVs recorded for the determination of TST in reference material of human urine (**A**,**B**) and wastewater samples purified in a sewage treatment plant (**C**,**D**): (**A**): (**a**) 10 µL of sample + 0.03, (**b**) as (**a**) + 0.03, (**c**) as (**a**) + 0.06 nM TST, (**B**): (**a**) 10 µL of sample + 0.2, (**b**) as (**a**) + 0.2, (**c**) as (**a**) + 0.4 nM TST, (**C**): (**a**) 1 mL of sample + 0.03, (**b**) as (**a**) + 0.03, (**c**) as (**a**) + 0.06 nM TST, and (**D**): (**a**) 1 mL of sample + 0.2, (**b**) as (**a**) + 0.2, (**c**) as (**a**) + 0.4 nmol L^−1.^ TST. The DPAdSV parameters: ΔE_A_ of 150 mV, t_m_ of 5 ms, ν of 175 mV s^−1^, E_Pb dep. and TST acc._ of −1.1 V and E_Pb dep. and TST acc._ of 120 s.

The high performance of the DPAdSV procedure at the pSPCE/PbNPs for TST determination makes it a great potential for the analysis of environmental and biological samples. Therefore, the practical ability of DPAdSV at the pSPCE/PbNPs was checked by the determination of TST in reference material of human urine and wastewater samples purified in a sewage treatment plant without any separation steps. The samples were spiked with a known concentration of TST standard solution and analyzed by the standard addition method. Table 2 presents the obtained results. The very low value of LOD (2.2 × 10^−12^ mol L^−1^) allows for the use of small sample volumes and multiple dilutions of the sample in the electrolyte solution (10 × dilution of wastewater and 1000 × dilution of urine, which contributes to minimizing the interference from the sample matrix). The coefficient of variation values obtained between 0.8 and 4.7% indicate very good repeatability of the signal. The recovery values were between 98.7 and 104.5%, which confirms a satisfactory degree of accuracy of the DPAdSV procedure at the pSPCE/PbNPs. The DPAdSVs registered during the determination of TST in real samples are shown in Figure 10. The HPLC/PDA was applied to compare the results of TST analysis in samples without preliminary preparation. However, the concentrations of TST were below the LOD and LOQ of HPLC/PDA. The calculated LOD and LOQ for the standard solution were 7.5 × 10^−8^ and 2.5 × 10^−7^ mol L^−1^, respectively.

## 4. Conclusions

In summary, in this study, for the first time, an electrochemically pretreated screen-printed carbon electrode modified with lead nanoparticles (pSPCE/PbNPs) was introduced for trace analysis of testosterone (TST). The pSPCE/PbNPs were characterized by cyclic voltammetry (CV), electrochemical impedance spectroscopy (EIS), scanning and transmission electron microscopy (SEM and TEM), and energy-dispersive X-ray spectroscopy (EDS). The electrochemical pretreatment of the SPCE surface and electrochemical modification with PbNPs reduce the charge transfer resistance, inhibit the electron transfer kinetics, and significantly increase the active surface area of the sensor, which is translated into a significant increase in the TST reduction peak current. The DPAdSV procedure using the pSPCE/PbNPs is a highly sensitive and selective method for the determination of TST. The use of the pSPCE/PbNPs allows obtaining a very low limit of TST detection (2.2 × 10^−12^ mol L^−1^) and wide linear ranges of the calibration graph (1.0 × 10^−11^–1.0 × 10^−10^, 1.0 × 10^−10^–2.0 × 10^−9^, and 2.0 × 10^−9^–2.0 × 10^−8^ mol L^−1^). The practical ability of DPAdSV at the pSPCE/PbNPs was successfully confirmed by the determination of TST in spiked reference material of human urine and wastewater samples purified in a sewage treatment plant without any separation steps. These findings suggest that it is a promising analytical electrochemical sensing procedure for TST analysis in environmental and biological samples. Furthermore, the advantage of the sensor is its portability, which is very promising for quick field analysis.

## Figures and Tables

**Table 1:** Comparison of techniques for analysis of TST.

Electrode	Method	Linear Range [mol L^−1^]	LOD [mol L^−1^]	Application	Ref.
SWNT-EPPGE	SWV	5.0 × 10^−9^–1.0 × 10^−6^	2.8 × 10^−9^	Urine	[2]
GCE/BiF + CTAB	SWAdSV	1.0 × 10^−9^–4.5 × 10^−8^	3.0 × 10^−10^	Pharmaceutical formulations, urine	[3]
HMDE	AdSV	1.0 × 10^−8^–7.3 × 10^−6^	5.0 × 10^−9^	Pharmaceutical formulations	[20]
MD/graphite	DPV	1.0 × 10^−8^–1.0 × 10^−6^	4.1 × 10^−8^	Saliva	[1]
MD/Graphene	DPV	1.0 × 10^−7^–1.0 × 10^−6^	6.7 × 10^−9^	Saliva	[1]
MD/CNTs	DPV	1.0 × 10^−10^–1.0 × 10^−6^	1.4 × 10^−11^	Saliva	[1]
MD/fullerene C_60_	DPV	1.0 × 10^−8^–1.0 × 10^−6^	1.5 × 10^−8^	Saliva	[1]
SPE/MIP	CV	3.5 × 10^−18^–3.5 × 10^−15^	3.5 × 10^−17^	Urine	[41]
PbFE (GCE/PbF)	SWAdSV	2.0 × 10^−8^–3.0 × 10^−7^	9.0 × 10^−9^	Urine	[19]
AuE/DMIP	SWV	1.0 × 10^−14^–1.0 × 10^−13^	1.0 × 10^−14^	Urine	[21]
GCE/CTAB	SWAdSV	1.0 × 10^−8^–7.0 × 10^−8^	1.2 × 10^−9^	Pharmaceutical formulations, urine	[4]
pSPCE/PbNPs	DPAdSV	1.0 × 10^−11^–1.0 × 10^−10^ 2.0 × 10^−10^–2.0 × 10^−9^ 2.0 × 10^−9^–2.0 × 10^−8^	2.2 × 10^−12^	Urine, wastewater	This work

SWNT-EPPGE—edge plane pyrolytic graphite electrode modified with single-walled carbon nanotubes; GCE/BiF + CTAB—glassy carbon electrode modified with bismuth film and cetyltrimethylammonium bromide; HMDE—hanging mercury drop electrode; MD/graphite—maltodextrin-modified paste electrode based on graphite; MD/graphene—maltodextrin-modified paste electrode based on grapheme; MD/CNTs—maltodextrin-modified paste electrode based on carbon nanotubes; MD/fullerene C_60_—maltodextrin-modified paste electrode based on fullereneC_60_; SPE/MIP—screen-printed electrode modified with molecularly imprinted polymer; PbFE—lead film electrode; AuE/DMIP—gold electrode modified with a double-layered molecularly imprinted polymer; GCE/CTAB—glassy carbon electrode modified with cetyltrimethylammonium bromide; pSPCE/PbNPs—electrochemically pretreated screen-printed carbon electrode modified with lead nanoparticles; SWV—square-wave voltammetry; SWAdSV—square-wave adsorptive stripping voltammetry; AdSV—adsorptive stripping voltammetry; DPV—differential-pulse voltammetry; CV—cyclic voltammetry; DPAdSV—differential-pulse adsorptive stripping voltammetry.

**Table 2:** The outcomes of TST determination in reference material of human urine and wastewater purified in a sewage treatment plant.

	TST Concentration [µmol L^−1^] ± SD (*n* = 3)				
Sample	Added	Found DPAdSV	Found in Electrochemical Cell	Coefficient of Variation * [%]	Recovery ** [%]
Purified wastewater	0.0003	0.000297 ± 0.000012	0.0000297 ± 0.0000012	4.05	99.0
	0.002	0.00201 ± 0.000026	0.000209 ± 0.0000017	0.80	100.5
RM of human urine	0.03	0.0296 ± 0.0012	0.0000296 ± 0.0000012	4.07	98.7
	0.02	0.209 ± 0.0017	0.000209 ± 0.0000017	1.29	104.5

* Coefficient of variation [%] = (SD × 100)/Found DPAdSV, ** Recovery [%] = (Found DPAdSV × 100)/Added.

## Data Availability

The data presented in this study are available on request from the corresponding author.
